# A COPD-like model of viral exacerbation: synergistic effects with smoke and poly I:C

**DOI:** 10.1186/1476-9255-10-S1-P34

**Published:** 2013-08-14

**Authors:** F Schlerman, J Kubera, M Fleming, K Nocka, C Williams

**Affiliations:** 1 Inflammation and Remodeling Research, Pfizer Cambridge Massachusetts, 02140, USA

## 

Viral exacerbations in patients with COPD can accentuate disease severity by increasing lung inflammation and decreasing lung function. Using a synthetic analog of double stranded RNA (Poly I:C ) in combination with cigarette smoke, we have found synergistic increases in total lung inflammation as well as other tissue biomarkers compared to Poly I:C alone. Female C57 BL/6 mice were exposed to room air or the smoke of 3R4F Kentucky Reference cigarettes twice daily for two weeks. On days 0, 3 and 7 animals were dosed with intranasal PBS or 50 ug of Poly I:C. On day 11 we observed significant increases in total inflammation, absolute macrophages and absolute neutrophils in animals receiving smoke+Poly I:C compared to Poly I:C alone. There was also a trend showing increased absolute lymphocyte counts with increases observed in both CD4+ and CD8+ T cells in animals receiving Poly I:C alone or in combination with smoke. Gene expression from whole lungs showed synergistic increases in the TH17 cytokines, IL-21, IL-22 compared to Poly I:C alone. In addition, increases in the expression of elastin in lung tissue may indicate the beginning of tissue damage which could potentially lead to increased alveolar space with longer exposure to smoke. This model of viral exacerbation has robust, reproducible inflammation, with an inflammatory cell influx that resembles inflammation in human COPD and has the potential to identify novel protein and gene expression biomarkers.

